# Comparative Evaluation of Conventional and Real-Time PCR Assays for the Epidemiological Surveillance and Molecular Characterization of *Leptospira* in Asymptomatic Stray Dogs

**DOI:** 10.3390/ani16213357

**Published:** 2026-10-24

**Authors:** Jelena Marić, Ivona Subić, Marija Stojiljković, Miloš Petrović, Vladimir Gajdov, Svetlana Nedić, Sonja Obrenović

**Affiliations:** 1 Veterinary Institute of the Republic of Srpska “Dr. Vaso Butozan”, 78000 Banja Luka, Bosnia and Herzegovina; jelena.maric@virs-vb.com (J.M.); ivona.subic@virs-vb.com (I.S.); 2 Veterinary Specialized Institute Nis, Dimitrija Tucovica 175, 18000 Nis, Serbia; milosvsinis@gmail.com; 3 Scientific Veterinary Institute Novi Sad, Rumenacki Put 20, 21113 Novi Sad, Serbia; vladimir.g@niv.ns.ac.rs; 4 Department of Reproduction, Fertility and Artificial Insemination, Faculty of Veterinary Medicine, University of Belgrade, Bul. Oslobodjenja 18, 11000 Belgrade, Serbia; svetlana.nedic@vet.bg.ac.rs; 5 Department of Infectious Animal Diseases and Bee Diseases, Faculty of Veterinary Medicine, University of Belgrade, Bul. Oslobodjenja 18, 11000 Belgrade, Serbia

**Keywords:** zoonoses, One Health, urinary *Leptospira* DNA detection, Sanger sequencing, microscopic agglutination test (MAT), phylogenetic analysis, Bosnia and Herzegovina

## Abstract

This study compared serological and molecular approaches for detecting *Leptospira* spp. using paired blood and urine samples from 124 asymptomatic stray dogs. Blood sera were screened via the microscopic agglutination test (MAT) for ten serovars. Urine samples were analyzed using four PCR assays: a commercial quantitative PCR (qPCR), a *lipL32* qPCR, a conventional *rrs* PCR, and a multiplex PCR (mPCR). Serological exposure was detected in 26 (20.97%) dogs, while commercial qPCR, *rrs* PCR, *lipL32* qPCR, and mPCR assays yielded positive results in 31 (25.00%), 34 (27.42%), 25 (20.16%), and 23 (18.55%) dogs, respectively. Compared with commercial qPCR, the *lipL32* qPCR, *rrs* PCR, and mPCR demonstrated Positive Percentage Agreement (PPA) values of 80.65%, 87.10%, and 67.74%, and Negative Percentage Agreement (NPA) values of 100.00%, 92.47%, and 97.85%. Nineteen urine samples were positive across all four PCR assays. Overall, 42.74% (53/124) of dogs had at least one positive serological or molecular finding, including nine additional conventional PCR-positive findings without reliable sequence confirmation. Sequencing and phylogenetic analysis of 24 samples identified *L. interrogans* and *L. borgpetersenii*, representing the first molecular characterization of *Leptospira* in Bosnia and Herzegovina. The findings demonstrate the complementary value of serological and molecular approaches for *Leptospira* surveillance while emphasizing the need for cautious interpretation of unconfirmed molecular findings.

## Simple Summary

Leptospirosis is a widespread bacterial disease that affects both animals and humans. Dogs can carry these bacteria without showing any symptoms, posing a potential health risk to humans and other animals. This study investigated exposure to *Leptospira* and the presence of its DNA in urine in apparently healthy stray dogs in Bosnia and Herzegovina by analyzing blood samples using serological methods and urine samples using four molecular methods. In the primary cohort, 53 of 124 dogs (42.74%) showed serological evidence of exposure to *Leptospira,* with molecular findings suggesting its presence in urine, or both, although some molecular findings were not confirmed by sequencing. By combining both serological and molecular techniques, a higher number of positive findings was obtained than by using any individual method alone. These findings highlight the presence of *Leptospira* exposure and DNA detection among apparently healthy stray dogs and underscore the value of integrating different diagnostic approaches for effective surveillance and control.

## 1. Introduction

Leptospirosis is a global zoonotic disease caused by pathogenic bacteria of the genus *Leptospira*. It is recognized as a major public health concern with significant potential for re-emergence. Although distributed worldwide, the disease is particularly prevalent in tropical and subtropical regions characterized by heavy rainfall and frequent flooding. While historically of primary importance in developing countries, the incidence of leptospirosis in temperate regions is expected to rise. This increase is driven by climate change, which causes extreme weather events and rising temperatures, as well as continuous urban expansion that intensifies contact between humans, domestic animals, and wildlife [1,2].

Numerous animals, including rodents (the primary reservoir), domestic mammals, and wildlife, serve as reservoirs for leptospires. Dogs play an important role in the epidemiology of *Leptospira* infection, acting as both incidental and maintenance hosts. They can shed the bacteria in their urine with or without displaying clinical signs, thereby contaminating the environment and posing a substantial risk to public health [1,3]. Because dogs are often more exposed to environmental infection sources than humans, they can serve as sentinels for environmental contamination or as potential epidemiological links between the environment and human populations [4]. Identifying circulating *Leptospira* serovars in dogs is essential for understanding local zoonotic risks. This is especially true for dogs with uncontrolled outdoor access, which easily contract the infection through contact with other animals or contaminated soil and water, subsequently excreting the pathogen in urine [3].

Various diagnostic methods are available to confirm leptospirosis. Although the microscopic agglutination test (MAT) remains the reference serological method, it exhibits several limitations. These include a strict dependence on a live antigenic panel, an inability to reliably distinguish between active infection, previous exposure, or vaccination, and cross-reactivity [5]. Furthermore, antibodies are typically undetectable during the first one to two weeks post-infection, hindering early diagnosis. MAT is also suboptimal for identifying chronic asymptomatic renal carriage and leptospiruria, as serum antibody titers do not necessarily correlate with shedding. Consequently, serological and molecular results are frequently discordant, and many infected shedding animals remain seronegative [6,7]. Nevertheless, the epidemiological value of MAT lies in its capacity to predict circulating serogroups [7]. To minimize false-negative results, the live antigen panel should include representative serovars of all locally circulating serogroups [5,8,9]. Conversely, bacterial isolation is highly specific but time-consuming (requiring 4 to 12 weeks), suffers from low diagnostic sensitivity, and is prone to contamination, which limits its utility in clinical and epidemiological studies [5,9].

Therefore, the complementary use of molecular methods is highly encouraged. The polymerase chain reaction (PCR) is a highly sensitive and specific molecular tool capable of detecting leptospiral deoxyribonucleic acid (DNA) in clinical and environmental samples [10]. PCR can detect leptospires in blood during the acute phase of infection, whereas urine samples are ideal during the late acute or chronic phases [6]. Numerous conventional and real-time PCR (qPCR) assays have been developed targeting either conserved genes like *rrs* (16S rRNA) [11] and *secY* and *flaB* genes [12], or genes encoding pathogenic outer membrane proteins such as *lipL32*, *lipL21*, and *lipL41* [13,14]. Among these, assays targeting the *lipL32* gene predominate. Because *lipL32* is a major outer membrane lipoprotein restricted to pathogenic species, its targeting allows for the selective detection of pathogenic *Leptospira* spp., thereby increasing assay specificity [5,10].

Conventional PCR assays offer the advantages of simpler execution and lower cost, making them suitable for routine diagnostics in resource-limited settings [11,15]. In contrast, quantitative real-time PCR (qPCR) assays provide faster detection, higher analytical sensitivity, and a significantly lower risk of cross-contamination because amplification and detection occur within a closed system, eliminating the need for post-PCR gel electrophoresis [10]. Notably, the serological classification into serovars and serogroups does not fully align with genetic classification; serovars of the same serogroup may belong to different genetic species, while a single genetic species can encompass multiple serovars [16]. Although both *lipL32* qPCR and conventional PCR assays are validated for rapid molecular diagnosis, studies systematically evaluating and comparing their diagnostic performance across different canine specimen types, including whole blood, serum, and urine, remain relatively limited. Furthermore, most available comparative research has focused on clinically ill dogs suspected of acute infection rather than asymptomatic, healthy dog populations evaluated alongside serological screening using the MAT test [15,17,18,19,20].

In a previous study focusing on the molecular detection of *Leptospira* spp. in the urine of asymptomatic stray dogs, a commercial qPCR assay with an undisclosed target gene revealed a high frequency of *Leptospira* DNA detection in urine [21]. This unexpected finding necessitated a secondary, thorough assessment of the observed molecular detection rate using alternative PCR approaches. Consequently, the present study employs three additional molecular methods alongside the commercial qPCR assay: an *rrs* (16S rRNA) conventional PCR assay, a multiplex PCR (mPCR) assay using the G1/G2 and B64-I/B64-II primer sets, and a *lipL32*-based qPCR assay [11,13,22]. Specifically, the primary objectives of this study are to (i) evaluate and compare the performance of these different PCR assays on urine samples and determine their mutual agreement; (ii) screen for anti-*Leptospira* antibodies using the microscopic agglutination test (MAT); and (iii) analyze the concordance between serological and molecular findings within this asymptomatic stray dog population. Furthermore, this study aims to perform species identification and phylogenetic analysis by sequencing selected *rrs*- and *secY*-positive amplicons to characterize the specific *Leptospira* spp. circulating among these animals.

## 2. Materials and Methods

### 2.1. Study Area

**Figure 1:**
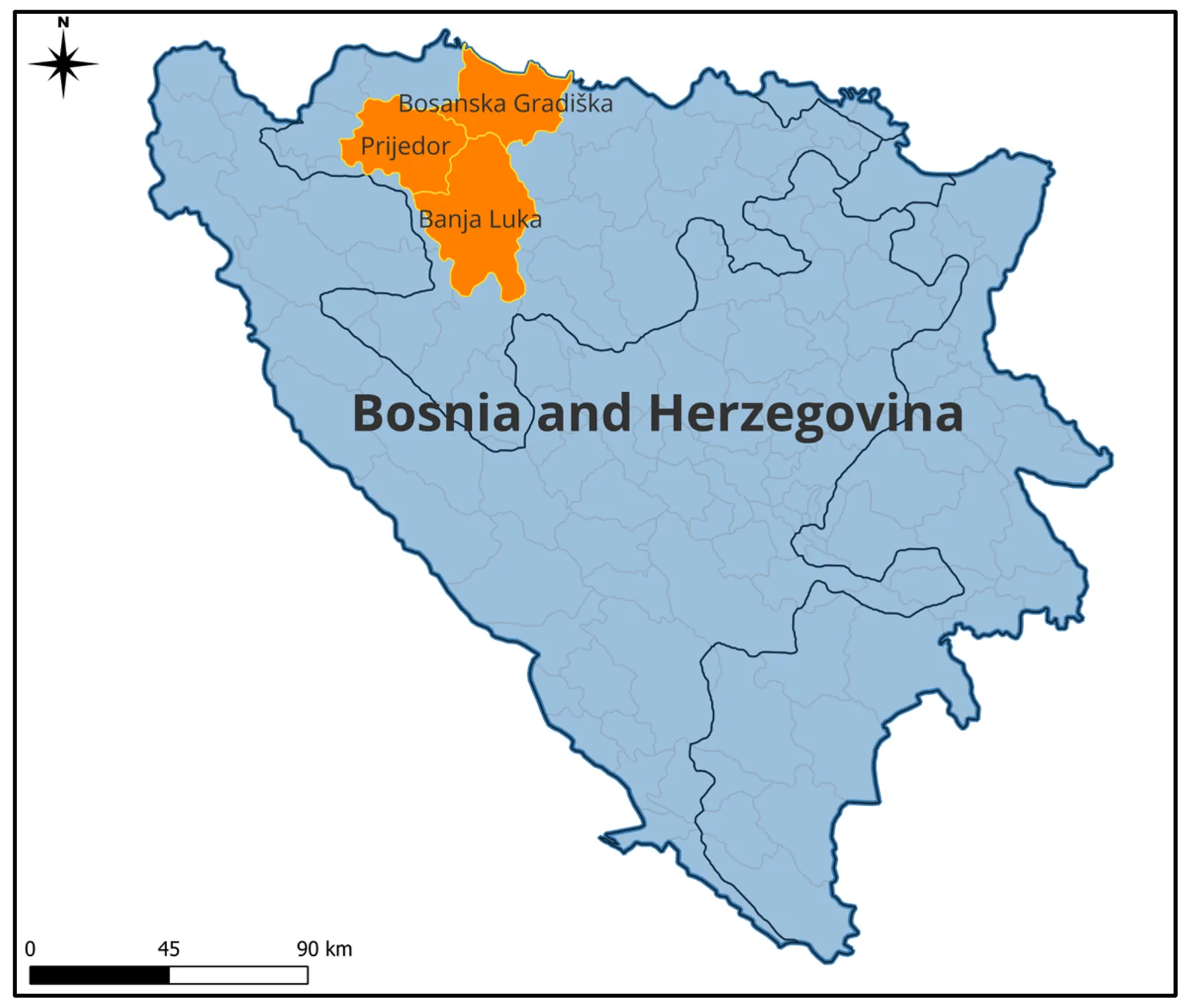
Geographical location of the study area in Bosnia and Herzegovina from which the examined stray dogs originated.

The study was conducted within a limited geographical zone in the northwestern part of Bosnia and Herzegovina (Entity of Republika Srpska), encompassing three neighboring municipalities (Banja Luka, Prijedor and Gradiška) (Figure 1), located approximately between 44°15′ and 45°15′ N latitude and 16°30′ and 18°15′ E longitude. The study region is predominantly characterized by a temperate continental climate, with moderately cold winters and warm summers. Climatic conditions vary somewhat across the territory due to differences in altitude and topography. Mean annual air temperatures generally range from 10 °C to 13 °C, while annual precipitation is typically 700–1050 mm, distributed throughout the year. Hydrographically, this region belongs to the Danube River Basin and features a dense network of waterways, including the Sava, Vrbas and Sana rivers. These river basins create extensive alluvial plains and seasonally flooded areas, providing potentially favorable environmental conditions for the environmental persistence and transmission of *Leptospira* spp. Due to the close proximity of these municipalities and their shared ecological features, the entire region was treated as a single regional study area.

### 2.2. Study Design and Sample Collection

This study builds upon the preliminary investigation described by Marić et al. [21], utilizing a primary study cohort of 124 asymptomatic stray dogs of varying sexes, breeds, and ages. No historical data regarding prior vaccinations, including immunization against leptospirosis, were available for the enrolled population. The total dataset comprises paired blood and urine samples structured to differentiate between newly analyzed animals and a previously screened subset. The newly enrolled cohort (*n* = 124) consists of 118 dogs sampled prospectively between July 2021 and November 2025 at local veterinary clinics and animal shelters operating under municipal stray dog population management programs. These programs follow the Catch–Neuter–Vaccinate–Return (CNVR) protocol, which includes mandatory surgical sterilization, microchipping, rabies vaccination, and antiparasitic treatment. Prospective sample collection was conducted in accordance with the guidelines and under the explicit approval of the relevant institutional ethics committee. Additionally, six dogs whose samples (two collected in 2018 and four in 2020) were retrieved from the laboratory archive were included. These archived samples had originally been collected for routine veterinary diagnostic purposes independently of the present research protocol and originated from asymptomatic dogs belonging to the same target population.

Separately, this study presents the results for a subset of 11 asymptomatic dogs that had tested positive by the commercial qPCR assay in our previous study (including one sample that was also positive by MAT). In the present study, these 11 samples underwent repeat commercial qPCR testing and additional molecular evaluations—specifically, conventional *rrs* PCR, conventional mPCR, *lipL32* qPCR, and subsequent sequencing—which had not been performed in the prior study. This separate evaluation of the previously confirmed samples was performed to achieve comprehensive phylogenetic characterization and assess assay agreement without confounding the primary study cohort. Primary statistical analyses of detection frequencies and inter-assay agreement were restricted to the 124 previously unpublished dogs. The 11 previously characterized samples were retained as a separate positive-enrichment panel for additional molecular testing and sequencing.

Dogs were classified as asymptomatic (i.e., clinically healthy) based on thorough clinical examinations conducted by veterinary staff upon admission to shelters or clinics for routine procedures. Upon admission, each animal was systematically scanned for a pre-existing microchip to minimize the possibility of repeatedly enrolling the same animal. Regarding urine collection, although explicit guidelines emphasizing aseptic catheterization were provided to all participating facilities, the collection method used for each individual sample could not be independently verified, and the possibility of free-catch collection under field conditions could not be excluded. Consequently, to maintain analytical integrity, the pooled results are presented strictly as a diagnostic positivity/detection rate rather than a representative population prevalence, and no sub-analysis based on the collection method was performed. No additional sampling or other procedures were performed on the six dogs represented by archived samples for the purposes of the present study.

Blood samples (2.0 mL) were collected aseptically via cephalic venipuncture into vacutainers without anticoagulants. The tubes were centrifuged at 2000× *g* for 10 min, and the separated serum was aliquoted and stored at −20 °C until serological analysis.

Approximately 5 mL of urine was collected from each dog into a sterile sample collection vial using urinary catheterization or, potentially, free-catch collection under field conditions, and stored at 4 °C. Each urine sample was processed for DNA extraction within 48 h of collection. Briefly, up to 2 mL of each urine sample was centrifuged at 14,000× *g* for 10 min, and the supernatant was discarded.

### 2.3. DNA Extraction and Amplification

Genomic DNA was extracted from the resuspended urine pellet in 180 µL Buffer ATL using the QIAamp^®^ DNA Mini Kit (Qiagen, Hilden, Germany), according to the manufacturer’s protocol “Isolation of bacterial DNA from biological fluids”. The extracted DNA was stored at −20 °C until further PCR analysis.

To detect *Leptospira* genomic DNA in the urine samples, four distinct molecular assays were employed: a commercial qPCR assay with an undisclosed target gene, a qPCR assay targeting the *lipL32* gene [13], a *rrs* PCR assay [11], and an mPCR assay [22].

The *rrs* (16S rRNA) conventional PCR assay was performed using the LeptoA and LeptoB primers (Eurofins Genomics Germany GmbH, Ebersberg, Germany) to amplify a 331-bp fragment of the *Leptospira rrs* gene, based on the method by Mérien et al. [11], with modifications. The PCR mixture was prepared in a final volume of 25 µL, containing 12.5 µL Biozym Red HS Taq Master Mix (Biozym Scientific GmbH, Hessisch Oldendorf, Germany), 1 µL of each primer (10 µM), 5 µL of template extracted DNA, and nuclease-free water to the final volume. DNA extracted from *L*. *interrogans* serovar Pomona was used as a positive control, while ultrapure water served as a negative control. Amplification was performed in a Labnet MultiGene Thermal Cycler (Labnet International, Edison, NJ, USA). The thermocycling profile began with an initial denaturation and enzyme activation step at 95 °C for 2 min. This was followed by an initial amplification cycle consisting of denaturation at 94 °C for 3 min, annealing at 63 °C for 90 s, and extension at 72 °C for 2 min, followed by an additional 34 cycles of denaturation at 94 °C for 1 min, annealing at 63 °C for 90 s, and extension at 72 °C for 2 min. The reaction concluded with a final extension step at 72 °C for 10 min, followed by cooling to 4 °C.

The multiplex PCR (mPCR) assay was performed using the G1/G2 and B64-I/B64-II primer sets originally described by Gravekamp et al. [12], with modifications based on their application to urine by Bal et al. [22]. This assay utilizes two primer sets specific for distinct groups of pathogenic *Leptospira* spp. The G1/G2 primers (Eurofins Genomics Germany GmbH, Germany) target the *secY* gene, amplifying a 285-bp fragment from strains belonging to *Leptospira interrogans*, *L. borgpetersenii*, *L. weilii*, *L. noguchii*, *L. santarosai*, and *L. meyeri* (strain ICF only). The B64-I/B64-II primers (Eurofins Genomics Germany GmbH, Germany) target the *flaB* gene, amplifying a 563-bp fragment specific to *L. kirschneri*. The sequences of all primers are detailed in Table 1.

The mPCR reaction mixture was prepared in a final volume of 25 µL, containing 12.5 µL Biozym Red HS Taq Master Mix (Biozym Scientific GmbH, Germany), 0.7 µL of each primer (10 µM), and 5 µL of template DNA and nuclease-free water to volume. DNA extracted from *L. interrogans* serovar Pomona and *L. kirschneri* serovar Grippotyphosa (strain Moskva V) were used as positive controls for the G1/G2 and B64-I/B64-II primer sets, respectively, while ultrapure water served as a negative control. Amplification was performed in a Mastercycler Nexus (Eppendorf SE, Hamburg, Germany). The thermocycling profile consisted of an initial Hot-Start enzyme activation at 95 °C for 2 min, followed by 37 cycles of denaturation at 94 °C for 90 s, annealing at 55 °C for 1 min, and extension at 72 °C for 2 min. The reaction concluded with a final extension step at 72 °C for 10 min, followed by cooling to 4 °C.

The conventional PCR products were analyzed by electrophoresis on a 1.5% agarose gel prepared in Tris-Borate-EDTA (TBE) buffer containing Midori Green Advance (5 µL per 100 mL of agarose gel) (Nippon Genetics Europe GmbH, Düren, Germany). The run was for 60 min at 100 V. A 100-bp DNA ladder was used to estimate fragment sizes (Qiagen, Hilden, Germany). The amplified products were visualized under UV light at 302 nm using a MacroVue UV-20 transilluminator (Hoefer Pharmacia Biotech, Bridgewater, MA, USA).

The *lipL32* qPCR assay was performed according to Stoddard et al. [13]. The reactions were carried out in a final volume of 25 µL, containing 12.5 µL of Brilliant III Ultra-Fast qPCR Master Mix (Agilent Technologies, Santa Clara, CA, USA), 0.2 µL of each primer (50 µM), 0.1 µL of the TaqMan probe *LipL32*-189P (50 µM) (Metabion International AG, Planegg, Germany), 3 µL of template DNA, 1 µL Bioline qPCR Extraction Control Red (Cy5) amplification reagents (Meridian Bioscience, London, UK), and nuclease-free water to volume. The final concentrations were 400 nM for each primer and 200 nM for the probe. DNA from the reference strain *L. interrogans* serovar Icterohaemorrhagiae and nuclease-free water were used as positive and negative controls, respectively. All reactions were performed in duplicate using the 7500 Fast Real-Time PCR System (Applied Biosystems, Foster City, CA, USA). An exogenous internal process control (Bioline qPCR Extraction Control Red) was added to each urine sample prior to DNA extraction and co-amplified with the *lipL32* target in the same qPCR reaction. The *lipL32* target was detected in the FAM channel, whereas the internal control was detected in the Cy5 channel. The established acceptable Ct range for the internal control was 27–30, and all samples yielded Ct values within this range. The amplification protocol included an initial denaturation at 95 °C for 3 min, followed by 45 cycles of denaturation at 95 °C for 15 s and annealing/extension at 60 °C for 60 s. Data were analyzed using the manufacturer’s software, and results were interpreted based on the cycle threshold (Ct) value. Samples with a Ct value ≤ 40.00 were considered positive. The sequences of all primers and probes are detailed in Table 1.

The commercial qPCR assay was performed using the Leptospira Pathogenic Real Time Kit (Microboss Hightech GmbH, Duisburg, Germany) following the manufacturer’s instructions. Each reaction mixture contained 2 μL of extracted genomic DNA and 18 μL of the provided master mix, yielding a total volume of 20 μL. According to the manufacturer’s standard package insert, the assay is designed to detect pathogenic *Leptospira* species; however, the specific target gene and the exact list of detectable species are not disclosed in the provided manual. Upon our specific inquiry, the manufacturer provided technical data confirming that the assay explicitly detects *Leptospira weilii*, *L. interrogans*, *L. borgpetersenii*, *L. noguchii*, *L. kirschneri*, *L. fainei*, *L. santarosai*, *L. johnsonii*, and *L. wolffii*, while the precise sequence of the target region remains proprietary. Positive and negative amplification controls were included in each amplification run to ensure assay validity. Amplification and detection were performed on the AriaMx Real-Time PCR System (Agilent Technologies, USA), and fluorescence data were analyzed using the instrument’s integrated software (version 2.0). The thermocycling profile consisted of an initial denaturation step at 95 °C for 15 s, followed by 45 cycles of denaturation at 95 °C for 15 s and annealing/extension at 60 °C for 60 s. To ensure maximum specificity and avoid potential false-positive signals in downstream statistical analyses, a conservative cycle threshold (Ct) cut-off of ≤40.00 was applied to define positive samples, whereas any amplification observed with a Ct value > 40.00 was strictly considered negative.

### 2.4. Assay Reproducibility, Quality Control, and Analytical Performance

All samples were tested in duplicate. In cases of discordant results between the initial replicates (one positive and one negative), the analysis was repeated in duplicate. A sample was classified as positive only when both replicates yielded positive results, either during initial testing or upon repeat testing. PCR-grade water was processed through the complete DNA extraction procedure alongside the samples as a negative extraction control, while nuclease-free water was used as a negative amplification control. Appropriate positive amplification controls were included for each assay, as described above.

To assess DNA extraction performance and potential PCR inhibition, an exogenous internal process control (Bioline qPCR Extraction Control Red, Cy5) was added to all urine samples before DNA extraction and subsequently co-amplified with the *lipL32* target in the same real-time PCR reaction, as detailed in Section 2.3. The established acceptable Ct range for the internal control was 27–30, and all samples yielded Ct values within this range. No evidence of substantial extraction failure or inhibition affecting the internal control reaction was observed. The commercial qPCR assay included the positive and negative amplification controls supplied with the kit, according to the manufacturer’s instructions.

The analytical characteristics of the assays were considered based on available validation data and previously published protocols. The commercial qPCR assay was performed using the manufacturer’s reagents, with the interpretation criteria described in Section 2.3. The *lipL32* qPCR assay had previously undergone in-house validation and is included in the laboratory’s scope of accreditation under ISO/IEC 17025:2017. The validation included assessments of repeatability, within-laboratory reproducibility, specificity, exclusivity, and robustness. Repeatability was evaluated in three runs of eight replicates, while within-laboratory reproducibility was assessed by two analysts performing eight replicates each. All replicates yielded positive results, corresponding to 100% qualitative agreement under the evaluated conditions. Exclusivity testing against 20 non-target microorganisms revealed no detectable amplification. Robustness was assessed using two real-time PCR platforms (ABI 7500 Fast software version 1.4.2.1, and QuantStudio 5, QuantStudio Design and Analysis Software version 1.5.1) and primer/probe concentration variations of ±10%, without changes in the qualitative results. The assay also demonstrated an amplification efficiency of 98.95% during laboratory performance assessment. The conventional *rrs* PCR and mPCR assays were based on published protocols [11,12,22], with modifications involving the PCR master mix and thermal cycling conditions, as described above. The analytical limits of detection of all four assays were not independently determined in a standardized side-by-side evaluation as part of the present study. Therefore, comparisons focused on observed detection frequencies and agreement between assays rather than absolute analytical sensitivity.

### 2.5. Sequencing and Phylogenetic Analysis

To achieve species-level identification and delineate the phylogenetic relationships of the circulating *Leptospira* spp. strains, 24 samples that tested positive via the commercial qPCR assay were selected for Sanger sequencing. Additionally, to evaluate amplification specificity and investigate potential false-positive or non-specific reactions, 5 samples that tested negative by the commercial qPCR but positive by conventional PCR assays were also submitted for sequencing. A total of 27 PCR products derived from the commercial qPCR-positive cohort yielded sequences suitable for downstream analysis, comprising 15 amplicons of the *rrs* gene and 12 amplicons of the *secY* gene; both target genes were successfully amplified and sequenced in three of these samples. In contrast, sequencing of the discordant, commercial qPCR-negative samples confirmed either non-specific amplification or yielded sequences of insufficient length and quality for reliable alignment. Consequently, these discordant samples, along with any other amplicons yielding non-specific or insufficient-quality sequences, were excluded from the final phylogenetic analyses.

The 331-bp fragment of the *rrs* gene (encoding 16S rRNA) was amplified using the *rrs* PCR assay described above, whereas the 285-bp *secY* gene fragment was amplified using the G1/G2 primer set as described for the mPCR assay above. Appropriate positive and negative controls were included in each amplification run under the assay-specific conditions previously detailed. The selected *rrs* and *secY* amplicons were submitted to Macrogen Europe (Amsterdam, Netherlands) for enzymatic purification and bidirectional (forward and reverse) Sanger sequencing. Sequencing reactions utilized the primary PCR primer pairs (LeptoA/LeptoB for *rrs* and G1/G2 for *secY*) at a final concentration of 10 pmol/µL. The raw chromatograms were inspected for quality, edited, and assembled into consensus sequences using the Staden Package (https://staden.sourceforge.net/) [23]. Sequence identification was performed by comparing the consensus sequences against reference sequences available in the GenBank database (https://www.ncbi.nlm.nih.gov/genbank/; accessed on 16 September 2026) via the BLASTn web interface (https://blast.ncbi.nlm.nih.gov/Blast.cgi; accessed on 16 September 2026). The phylogenetic reconstruction methods were selected according to the objectives of the analyses and the characteristics of the two genetic markers. The highly conserved *rrs* gene was analyzed using the Neighbor-Joining (NJ) method to assess the broad phylogenetic placement of the obtained sequences within the genus *Leptospira*. In contrast, the more variable protein-coding *secY* gene was analyzed using Maximum Likelihood (ML) under the Tamura 3-parameter substitution model to examine phylogenetic relationships among closely related pathogenic *Leptospira* species. Bootstrap analysis with 1000 replicates was performed for both phylogenetic reconstructions. All phylogenetic analyses, sequence alignments, and tree reconstructions were performed using the MEGA software (version 12). All nucleotide sequences generated in this study have been deposited in GenBank under the following accession numbers: PZ058895–PZ058909 for the *rrs* gene and PZ095795–PZ095806 for the *secY* gene.

### 2.6. Microscopic Agglutination Test (MAT)

Dog serum samples (n = 124) were analyzed using the microscopic agglutination test (MAT) in accordance with the guidelines of the World Organisation for Animal Health [24]. The MAT panel comprised ten reference serovars representing eight serogroups of *Leptospira* spp., sourced from the National Collaborating Centre for Reference and Research on Leptospirosis (Royal Tropical Institute, Amsterdam, the Netherlands) (Table 2). Serovar selection was informed by regional epizootiological data regarding their prevalence in dogs and wild carnivores [21,25]. A cut-off titer of ≥1:100 was established as the threshold for seropositivity. Samples identified as positive during this initial screening were subsequently evaluated using a complete series of two-fold serial dilutions, starting from 1:100 up to a maximum dilution of 1:12,800, with all positive samples titrated through the full dilution series, irrespective of the endpoint titer. The final titer was defined as the highest serum dilution demonstrating ≥50% agglutination of leptospires.

### 2.7. Statistical Analyses

Primary descriptive statistics and all inter-assay agreement analyses were restricted to the primary analytical cohort of 124 previously unpublished dogs. The 11 previously characterized samples were excluded from these calculations because they had been intentionally selected based on prior commercial qPCR positivity. Descriptive statistics were used to express the results of serological and molecular assays as percentages with associated 95% confidence intervals (CI), calculated using the Wilson score method. The seropositivity rate was calculated as the proportion of MAT-positive dogs relative to the total number of examined animals. The overall proportion of dogs with at least one positive finding was interpreted as evidence of serological exposure and/or *Leptospira* DNA detection in urine rather than active infection prevalence. Cohen’s kappa coefficient (κ) was utilized to evaluate the agreement between the different molecular assays, as well as between molecular and serological (MAT) results. The strength of agreement was interpreted according to Landis and Koch [26]. The exact McNemar test for paired samples was applied to assess systematic differences in the detection frequencies between the individual PCR assays and between MAT and the commercial qPCR assay. The commercial qPCR assay was selected as the baseline comparator to evaluate the relative performance of the other molecular tools (*rrs*, mPCR, and *lipL32* assays). Because an independent molecular gold standard was unavailable and the commercial assay’s target sequence is proprietary, the comparative results are presented as Positive Percentage Agreement (PPA), Negative Percentage Agreement (NPA), and Overall Percentage Agreement (OPA), rather than absolute diagnostic sensitivity, specificity, and accuracy. Discordant results between assays were interpreted as disagreements in detection rather than definitive false-positive or false-negative findings. All statistical analyses were performed using MedCalc Statistical Software version 22.0 (MedCalc Software Ltd., Ostend, Belgium), with statistical significance set at *p* < 0.05.

## 3. Results

### 3.1. Molecular Detection via PCR Assays

In the primary analytical cohort of 124 previously unpublished dogs, the commercial qPCR assay detected *Leptospira* DNA in 31 urine samples (25.00%; 95% CI: 18.21–33.29%). The *lipL32* qPCR assay yielded 25 positive results (20.16%; 95% CI: 14.05–28.07%), all of which were also positive by commercial qPCR.

The mean Ct values were 33.75 (range: 25.40–37.45) for *lipL32* and 33.90 (range: 28.00–38.83) for the commercial qPCR. The conventional *rrs* PCR assay detected 34 positive samples (27.42%; 95% CI: 20.34–35.86%), while mPCR yielded 23 positive results (18.55%; 95% CI: 12.69–26.30%), all obtained using the G1/G2 primer set, whereas none yielded an *L. kirschneri*-specific amplicon with the B64-I/B64-II primer set.

One sample showed late amplification by the commercial qPCR assay (Ct 44.97). Although this result was considered borderline according to the manufacturer’s technical manual, it was classified as negative based on our study’s conservative criteria (Ct ≤ 40.00) and was excluded from the final commercial qPCR-positive group. Interestingly, this borderline sample tested positive via the conventional *rrs* PCR and was subjected to sequencing; however, a sequence of satisfactory quality could not be recovered, possibly due to a low concentration of target DNA. The separate panel of 11 previously characterized samples yielded ten positive commercial qPCR results upon retesting. Seven samples were positive by *rrs* PCR, five by mPCR, and five by *lipL32* qPCR. One sample, previously commercial qPCR-positive, yielded a borderline Ct value of 44.34 and was classified as negative in the present study, thereby further illustrating the challenges of interpreting borderline results in samples with low DNA concentrations. This sample was MAT-positive for serovars Pomona (1:400) and Australis (1:100), while all other PCR assays yielded negative results. Among the remaining ten commercial qPCR-positive samples, 70.00% (7/10; 95% CI: 39.68–89.22%) were positive by *rrs* PCR, and 50.00% (5/10; 95% CI: 23.66–76.34%) by each of the mPCR and *lipL32* qPCR assays. The mean Ct values were 33.77 (range: 29.38–37.85) for commercial qPCR and 33.31 (range: 31.20–35.17) for *lipL32* qPCR. Comparative serological and molecular findings for all samples with at least one positive result are summarized in Table 3.

Among the 31 samples that tested positive by the commercial qPCR assay, 27 (87.10%; 95% CI: 71.15–94.87%) were concurrently positive by the *rrs* PCR, 21 (67.74%; 95% CI: 50.14–81.43%) by the mPCR, and 25 (80.65%; 95% CI: 63.72–90.81%) by the *lipL32* qPCR. Interestingly, among the 93 samples that tested negative by the commercial qPCR, seven tested positive via the conventional *rrs* PCR and two via the mPCR. Consequently, conventional PCR methods identified a total of nine additional PCR-positive findings in samples negative by the commercial qPCR assay; however, these findings lacked reliable sequence-based confirmation (Table 3). The molecular assays demonstrated substantial overall concordance, with Kappa values (κ) ranging from 0.72 to 0.86 (Table 4). Compared to the commercial qPCR, the *rrs* PCR demonstrated the highest PPA (87.10%), while the *lipL32* qPCR achieved the highest NPA (100.00%). Systematic pairwise comparisons via the exact McNemar test revealed significant variations in detection frequencies between specific assays (*p* < 0.05), with 19 samples (15.32%) testing positive across all four molecular methods. All detailed agreement metrics and statistical comparisons are compiled in Table 4.

### 3.2. Results of Phylogenetic Analysis

**Figure 2:**
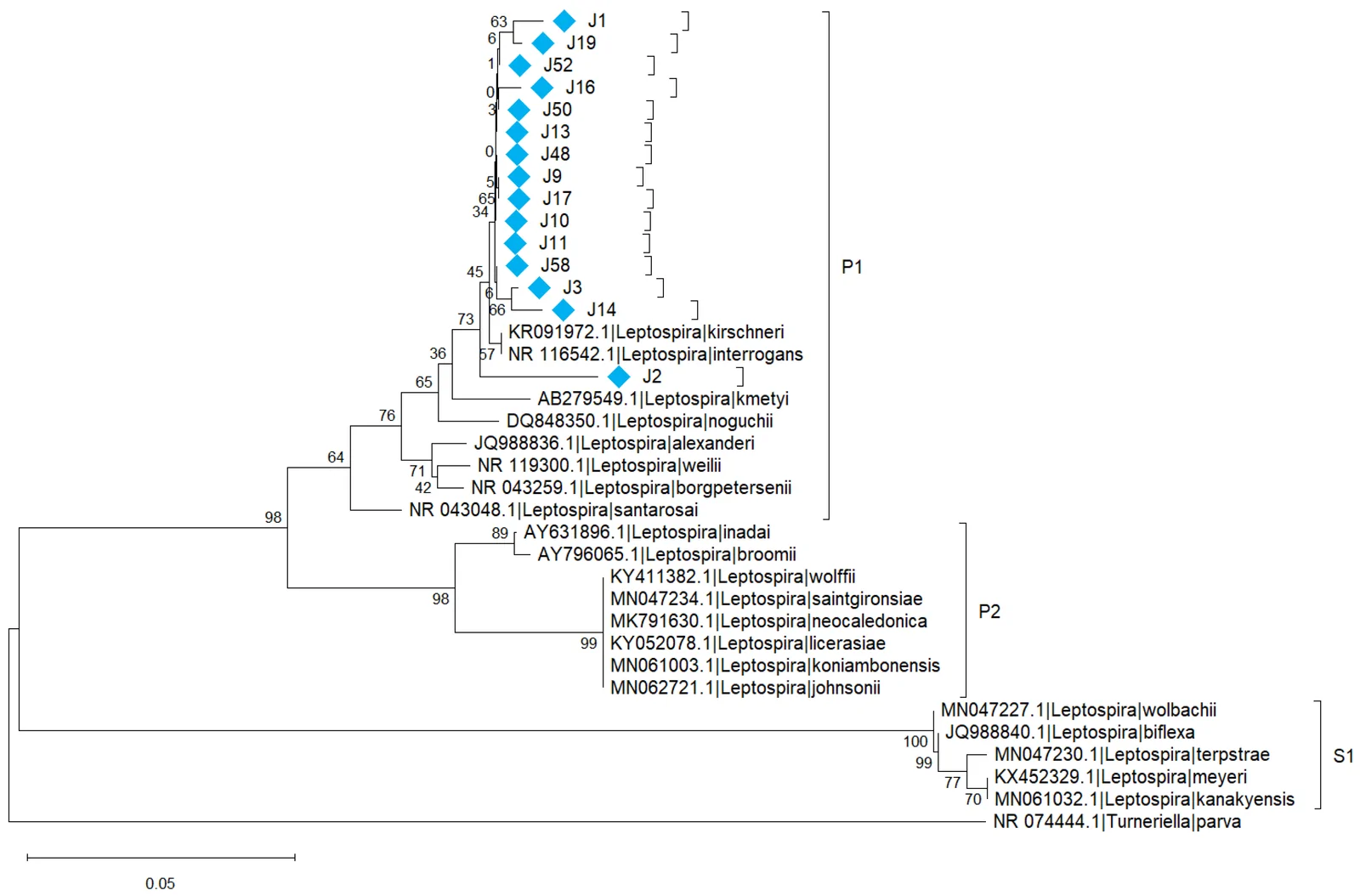
The *rrs-*based phylogenetic analysis of the *Leptospira* sequence types generated in this study and representative sequences of different species within the genus *Leptospira*. The blue diamonds indicate the original sequences obtained from canine urine samples in the current study, while the labels (e.g., J1, J19, J52) represent the unique laboratory identification codes assigned to the corresponding samples. The phylogenetic tree was inferred using the Neighbor-Joining method. The percentage of replicate trees in which the associated taxa clustered together in the bootstrap test (1000 replicates) is shown next to the branches.

**Figure 3:**
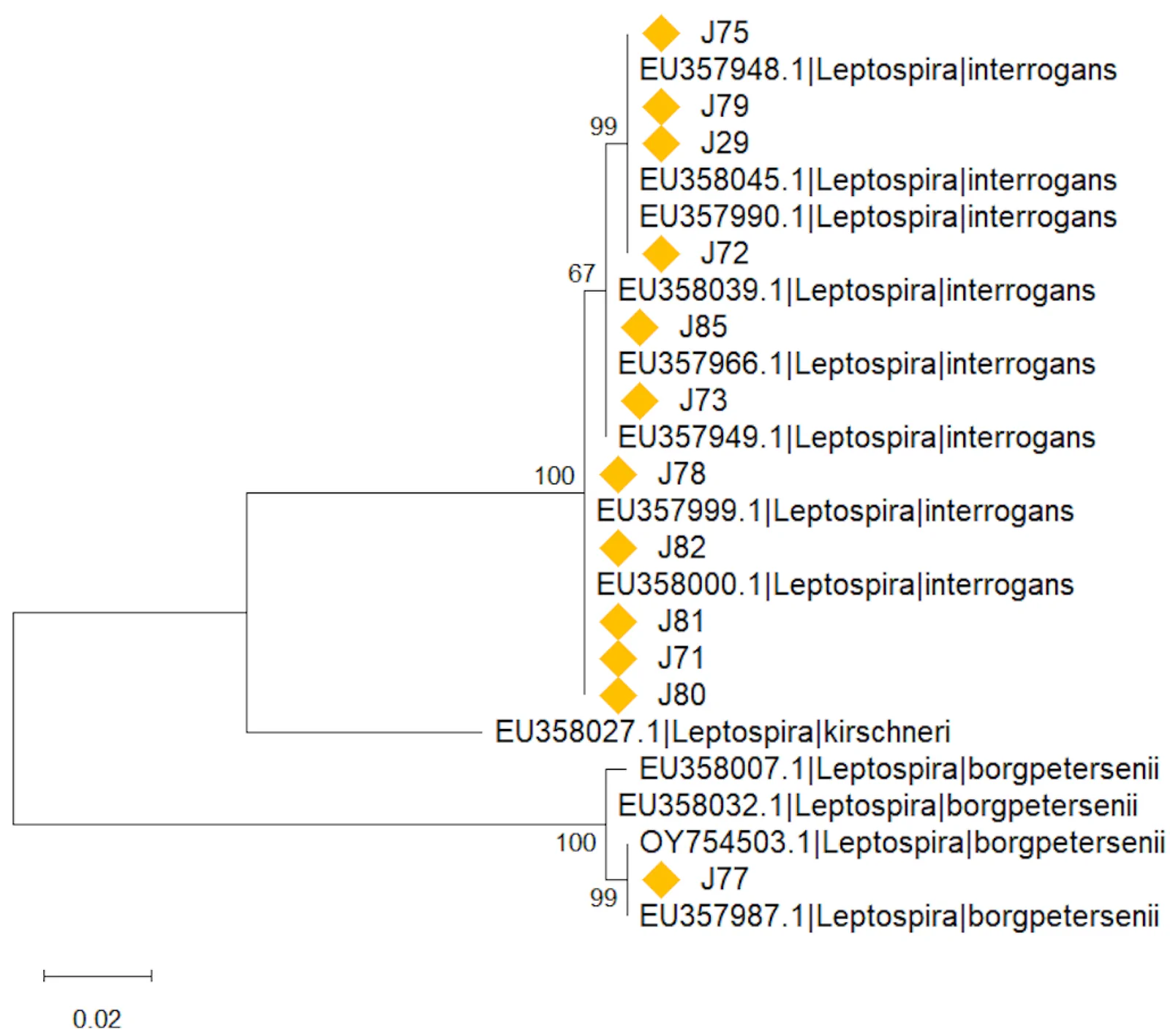
The *secY-*based phylogenetic analysis of the *Leptospira* sequences detected in this study and representative reference sequences. The yellow diamonds indicate the specific sequences generated from canine urine samples in this study, while the labels (e.g., J75, J79) represent their unique laboratory identification codes. The phylogenetic tree was inferred using the Maximum Likelihood method based on the Tamura 3-parameter model. Statistical support for the internal branches of the tree was evaluated by bootstrapping with 1000 replicates.

Phylogenetic analyses based on partial *rrs* (16S rRNA) and *secY* gene sequences are presented in Figure 2 and Figure 3. These analyses were performed to determine the taxonomic affiliation and genetic relationships of the *Leptospira* sequences detected in canine urine samples. Among the 24 samples with sequence-confirmed pathogenic *Leptospira* DNA, 17 belonged to the primary analytical cohort and seven to the previously characterized positive-enrichment panel.

Sequencing of the *rrs* and *secY* amplicons yielded high-quality chromatograms with nucleotide sequence identities equal to or greater than 99% compared to reference database entries. Among the *rrs*-positive samples, the BLASTn results [27] demonstrated that all recovered sequences clustered within the clade of pathogenic *Leptospira* species and were most closely related to members of the *L. interrogans* group. However, species-level identification based on partial *rrs* sequences should be interpreted with caution due to the limited resolution of this genetic marker among closely related pathogenic *Leptospira* species. Phylogenetic reconstruction based on the alignment of the *rrs* sequences successfully resolved the three main clades representing the pathogenic, intermediate, and saprophytic *Leptospira* species (Figure 2). Conversely, sequencing of the *secY* amplicons provided higher resolution, revealing that 11 sequences shared 99–100% identity with *L. interrogans*, while one sequence exhibited 99% identity with *L. borgpetersenii* (Figure 3). All generated sequences were unequivocally classified within the pathogenic *Leptospira* group, with the observed clustering topology strongly supported by bootstrap analysis.

A total of five samples that tested negative by the commercial qPCR but positive by conventional PCR assays, specifically three by the conventional *rrs* PCR and two by the conventional mPCR, were submitted for sequencing. However, these did not yield reliable data for further analysis. Among the *rrs* PCR samples, two produced non-specific sequencing results, and one yielded a sequence of unsatisfactory quality. For the two mPCR samples, one resulted in a non-specific sequence and the other was of unsatisfactory quality. Consequently, these five samples were excluded from the final phylogenetic analyses, as reliable species identification was not possible.

### 3.3. Serological Results via Microscopic Agglutination Test (MAT)

The serological exposure rate determined by MAT was 20.97% (26/124; 95% CI: 14.73–28.95%) at titers ≥1:100. The most frequently observed endpoint titer was 1:100 (9/26; 34.62%), followed by 1:400 (7/26; 26.92%), 1:200 (5/26; 19.23%), 1:800 (3/26; 11.54%), and 1:1600 (2/26; 7.69%). The titer of 1:1600 was the maximum titer detected in this study, and no samples demonstrated higher antibody levels up to the maximum tested dilution of 1:12,800. Serological reactivity was dominated by serovars belonging to the Australis serogroup. Specifically, antibodies against serovar Australis were detected in 13/26 seropositive dogs (50.00%; 95% CI: 32.06–67.94%), four of which concurrently reacted to serovar Bratislava from the same serogroup (4/26; 15.38%; 95% CI: 6.15–33.53%). Reactivity to other serovars was observed as follows: Pomona in 11 dogs (42.31%; 95% CI: 25.54–61.05%), Icterohaemorrhagiae in seven (26.92%; 95% CI: 13.70–46.08%), Canicola in four (15.38%; 95% CI: 6.15–33.53%), Grippotyphosa in three (11.54%; 95% CI: 4.00–28.98%), and Sejroe in one dog (3.85%; 95% CI: 0.68–18.89%). No reactivity was observed against serovars Bataviae, Autumnalis, or Hardjo. These findings represent overall MAT reactivity and do not confirm individual infecting serovars due to potential cross-reactivity.

Reactivity to a single serovar was observed in 14 dogs, while nine, one, and two dogs reacted to two, three, and four serovars, respectively. These results describe serological reactivity to the tested antigens and do not establish the infecting serovar.

Among the 31 commercial qPCR-positive dogs, 13 (41.94%; 95% CI: 26.42–59.23%) were MAT-positive, and 18 (58.06%; 95% CI: 40.77–73.58%) were MAT-negative. Of the 93 commercial qPCR-negative dogs, 13 (13.98%; 95% CI: 8.35–22.46%) were MAT-positive, and 80 (86.02%; 95% CI: 77.54–91.65%) were MAT-negative. The overall percentage agreement was 75.00%, with Cohen’s κ = 0.30, indicating fair agreement. The exact McNemar test showed no statistically significant difference in the proportions of positive results between MAT and commercial qPCR (*p* = 0.4731).

Overall, 44/124 dogs (35.48%; 95% CI: 27.61–44.23%) were positive by MAT and/or commercial qPCR. Collectively, when incorporating the results of all diagnostic assays used in this study (MAT, commercial qPCR, *lipL32* qPCR, and the conventional PCR assays), 53 out of 124 dogs (42.74%; 95% CI: 34.38–51.54%) displayed at least one positive result indicating serological exposure, molecular detection, or both. This cumulative total included nine additional dogs that tested negative by both MAT and commercial qPCR but positive by conventional PCR assays (7 dogs positive exclusively by the conventional *rrs* PCR and 2 dogs positive exclusively by the conventional mPCR); these nine findings lacked reliable sequence-based confirmation. This cumulative finding indicates aggregate serological exposure and/or molecular findings suggestive of *Leptospira* DNA presence in urine and should not be interpreted as a direct measure of active infection prevalence.

## 4. Discussion

Dogs play an important role in the epidemiology of leptospirosis, serving as either incidental or maintenance hosts that can shed *Leptospira* in their urine without displaying clinical symptoms. Given that stray dogs are highly vulnerable to pathogenic *Leptospira* due to frequent contact with diverse environmental and wildlife sources, understanding the epidemiological dynamics within these populations is essential for designing effective measures to mitigate transmission risks to the environment, other animals, and humans [21,28,29,30]. The choice of diagnostic modalities inevitably depends on local availability, cost, and laboratory feasibility, with each method presenting distinct advantages and limitations. Although serological surveys effectively estimate exposure rates to *Leptospira* spp., renal carriage and urinary shedding are not always accompanied by detectable antibody titers, thereby limiting the utility of serology in identifying asymptomatic shedders [6,17,18]. Consequently, the detection of leptospiral DNA via PCR is increasingly advocated as a valuable tool for investigating possible renal carriage [17,18,19]. However, variations in target genes, primer design, extraction protocols, amplification conditions, and interpretation thresholds across different PCR assays can significantly influence overall detection rates [5]. While a positive PCR result confirms the presence of leptospiral DNA, a negative result cannot definitively rule out infection due to transient leptospiremia and intermittent urinary excretion. To overcome these diagnostic hurdles, the accurate identification of chronically infected animals should ideally rely on prospective studies incorporating longitudinal, repeated sampling and multiple diagnostic methods. Ultimately, molecular results must always be interpreted with caution and evaluated in conjunction with serological findings [5,8].

In a previous preliminary study evaluating the urinary detection of *Leptospira* DNA in asymptomatic dogs, we employed a commercial qPCR assay with an undisclosed target gene and reported a molecular detection rate of 22% [21]. Utilizing the identical assay on the same sample type from dogs in the same geographic area, the present study determined a highly consistent molecular detection rate of 25.00%. Although that earlier study evaluated a smaller sample size, both investigations involved stray dogs from the same geographical region, suggesting that *Leptospira* DNA was detected in this population during different sampling periods. These molecular detection rates are notably higher than those reported across various European countries, which typically range from 0.2% to 8% [18,31,32,33]. Conversely, our findings align closely with the molecular detection rates of 20% to 48.8% observed in dogs from known endemic regions [4,34]. Although direct cross-study comparisons must be interpreted cautiously due to variations in dog populations, epidemiological contexts, sampling frequencies, PCR assays, and interpretation criteria, these findings warrant further investigation. They indicate a relatively high frequency of *Leptospira* DNA detection in urine from asymptomatic dogs in an area characterized by a temperate continental climate and moderate precipitation during the study period. Given that the utilization of multiple gene targets can help differentiate between true target amplification and non-specific PCR results [35], the complementary use of assays with defined genetic targets was considered valuable for investigating discordant molecular findings [11,13,22]. Furthermore, to evaluate the specificity of the obtained PCR products and identify the circulating *Leptospira* spp., sequencing and phylogenetic analyses of selected positive amplicons were performed.

Crucially, sequence analysis of the *rrs* (16S rRNA) and *secY* genes from samples positive by the commercial qPCR assay confirmed that the sequenced urine samples contained DNA belonging to pathogenic *Leptospira* spp., providing robust molecular evidence of pathogenic *Leptospira* DNA in urine. While establishing the absolute analytical detection limit of the assay was not possible without a direct side-by-side comparison of detection limits using precisely quantified reference material, the commercial qPCR detected samples with high Ct values, suggesting low target DNA concentrations, as reflected by Ct values spanning from 28.00 to 38.83 (Table 3). Furthermore, two borderline samples (one from the present study and one from the previous study) with late amplification profiles (Ct = 44.97 and Ct = 44.34) were observed and, according to the study’s predefined Ct cut-off (≤ 40.00), were classified as negative; subsequent attempts to sequence one of these samples failed, possibly due to a low target DNA concentration, resulting in an incomplete sequence.

The selection of the commercial qPCR assay as the baseline comparator was supported by its previous application in our study population [21]. Sanger sequencing and phylogenetic analysis provided additional molecular confirmation of pathogenic *Leptospira* DNA in 24 selected commercial qPCR-positive urine samples from the complete analytical dataset. However, sequencing was performed only on a selected subset; furthermore, the commercial assay cannot be regarded as an independent diagnostic gold standard. Therefore, inter-assay agreement analyses were restricted to the 124 previously unpublished dogs and were expressed as PPA, NPA, and OPA rather than absolute diagnostic sensitivity and specificity. When comparing the two real-time qPCR assays, almost perfect agreement was observed (κ = 0.86). Although the *lipL32* qPCR achieved an NPA of 100%, its PPA was 80.65%, yielding negative results in six samples positive by the commercial qPCR assay. Pathogenic *Leptospira* DNA was confirmed by sequencing in one of these six discordant samples; two additional sequence-confirmed discordant samples belonged to the separate positive-enrichment panel. These discrepancies may reflect differences in limit of detection, target copy number, reaction chemistry, or template concentration near the assay’s detection limit. This is consistent with previous literature, where modifying the original *lipL32* qPCR protocol by increasing the working concentration of primers and probes in clinical specimens achieved a ten-fold increase in analytical sensitivity, illustrating that modifications in reaction conditions may influence analytical sensitivity [36]. Globally, data regarding the analytical sensitivity of various qPCR assays in canine clinical matrices remain scarce, and several molecular protocols, including certain *lipL32* configurations, have yet to be fully validated across diverse canine sample types [19,20,37].

A comparison between the *rrs* PCR and *lipL32* qPCR assays demonstrated substantial agreement (κ = 0.76). Ten samples were *rrs*-positive but *lipL32* qPCR-negative, whereas one sample showed the opposite result. Sequence analysis confirmed pathogenic *Leptospira* DNA in one of the ten *rrs*-positive/*lipL32*-negative samples, while two additional sequence-confirmed discordant samples belonged to the separately analyzed positive-enrichment panel. The *rrs* PCR assay yielded positive results in seven samples classified as negative by commercial qPCR, whereas four samples were positive exclusively by commercial qPCR, resulting in 11 discordant findings. One of the seven *rrs*-positive/commercial qPCR-negative samples displayed a late amplification profile (Ct > 40) in the commercial qPCR, whereas the remaining six were positive exclusively via the *rrs* PCR assay. The successful detection by the *rrs* PCR in the sample with a late Ct (>40) profile may reflect a low concentration of target DNA near the assay’s detection threshold, where stochastic variation in amplification may occur. The presence of two copies of the *rrs* gene in pathogenic *Leptospira* genomes may contribute to differences in detection between assays; however, the target gene of the commercial qPCR assay is undisclosed, preventing a definitive explanation of the observed discordance. Sequencing was attempted for selected discordant *rrs* amplicons; however, the resulting sequences were either non-specific or of insufficient quality for species identification, indicating that non-specific amplification cannot be excluded as a possible contributor to the observed discordance.

When comparing the two conventional PCR assays, a moderate agreement (κ = 0.57) was observed, along with notable variations in agreement metrics. The mPCR demonstrated a lower PPA (67.74% vs. 87.10%) but a higher NPA (97.85% vs. 92.47%) compared to the *rrs* PCR. All positive mPCR outcomes were obtained using the G1/G2 primer set targeting the *secY* gene, with no amplification obtained using the B64-I/B64-II primer set. Prior studies have highlighted that the G1/G2 primer pair exhibits high specificity for pathogenic *Leptospira* while successfully excluding saprophytic strains; it has often shown superior overall sensitivity and specificity compared to assays targeting the *rrs*, *lipL32*, or *flaB* genes, making it a critical molecular target [9,15]. However, our findings indicate that the mPCR assay was not entirely free of diagnostic limitations, demonstrating a high NPA but lower PPA relative to the other methods. Although previous studies have associated limitations in mPCR detection and reproducibility with DNA extraction efficiency or co-extracted PCR inhibitors [15,22], a low target DNA concentration near the detection limit may also have contributed to discordant findings in our study.

Our findings align with previous studies where *rrs*-based PCR systems exhibited superior PPA but slightly lower NPA compared to *lipL32* qPCR assays [35,38,39]. Conversely, they contrast with reports that documented higher PPA for *lipL32* qPCR assays [19,28]. The different genomic copy numbers of the *rrs* and *lipL32* targets may contribute to differences in detection, although the present study did not independently establish their relative analytical limits of detection [35,38]. Furthermore, the fact that these two assays target entirely different genetic loci may account for some of the observed diagnostic discrepancies [15]. Additionally, the mPCR and the *lipL32* qPCR assay also demonstrated substantial agreement (κ = 0.74). The mPCR assay detected two samples that were negative by all other molecular assays; however, sequencing of one of these amplicons revealed non-specific amplification, whereas the other was not successfully characterized by sequencing.

However, for over two decades, the diagnostic specificity of *rrs* and mPCR assays has been scrutinised. These assays are prone to non-specific amplification of DNA from commensal or environmental microflora, which underscores why sequencing of positive amplicons is strongly recommended [13,15,19,20,37]. Furthermore, the urine collection method and the presence of contaminating lower urinary tract microbiota can contribute to non-specific amplification, as the ubiquitous presence of environmental and skin bacteria on the animal’s coat can easily compromise sample integrity. For instance, Fink et al. [19] reported high assay specificity when samples were obtained via catheterisation; conversely, non-specific amplification was observed in 13.3% of dogs whose urine was collected via free-catch urination. Although the participating institutions were provided with explicit guidelines regarding the proper collection of urine samples, including aseptic catheterization and collection of midstream urine into sterile containers, strict adherence to these procedures under all field conditions could not be independently verified. Nevertheless, stringent sample transport conditions, together with rigorous criteria for PCR detection and sequencing, were intended to reduce the risk of contamination and non-specific findings in the conventional PCR analyses. Additionally, conventional PCR workflows are inherently more susceptible to amplicon carryover contamination than closed real-time PCR systems. Thus, some of the observed discrepancies could stem from laboratory contamination and/or sample handling. In our study, nucleotide sequence analysis of amplicons derived from samples that tested positive exclusively by a single conventional PCR assay revealed similarity to *Citrobacter* spp. and *Escherichia coli.*

In the present study, out of the 31 commercial qPCR-positive samples, only 19 were simultaneously amplified across all four PCR assays. This indicates that targeting multiple genes and employing diverse molecular methods can provide complementary findings for the interpretation of *Leptospira* DNA detection, particularly in specimens with low target DNA concentrations [35,38]. High Ct values suggest low target DNA concentrations in some samples, potentially contributing to inconsistent amplification near the assay’s detection threshold. Additionally, we observed reproducibility challenges in a subset of positive samples, where *Leptospira* DNA could not be consistently detected across all replicates of the same sample. Such inconsistent amplification may arise from stochastic template distribution at low copy numbers. Discordant duplicate results were retested, and positivity required two positive replicates, as described in Section 2.4. Supporting these observations, one sample from the previous study that was initially positive by commercial qPCR yielded a negative result upon retesting, while the remaining molecular assays were also negative, as described above. This discrepancy may reflect a low concentration of target DNA, resulting in stochastic amplification and variable detection between repeated analyses, particularly near the assay’s detection limit.

Consequently, while conventional PCR assays remain reliable when paired with amplicon sequencing, the rapid turnaround time, closed amplification environment, and lower susceptibility to laboratory contamination position real-time qPCR platforms, such as the *lipL32* qPCR, as more practical tools for routine screening and epizootiological surveys. Interestingly, although the commercial qPCR assay in our study demonstrated a high molecular detection rate, its exact target gene remained undisclosed by the manufacturer. Subsequent inquiries to the manufacturer revealed that the assay is designed to detect a broad spectrum of *Leptospira* species, including *L. weilii*, *L. interrogans*, *L. borgpetersenii*, *L. noguchii*, *L. kirschneri*, *L. fainei*, *L. santarosai*, *L. johnsonii*, and *L. wolffii*. This expansive detection range suggests that the assay might facilitate the detection of a wide diversity of *Leptospira* strains, including intermediate species, although species-level identification requires additional molecular characterization. This is epidemiologically relevant, as intermediate *Leptospira* species have been previously detected in both humans and dogs [40,41]. However, a critical limitation within veterinary diagnostics is that most commercially available PCR assays exhibit variable analytical validation, often coupled with minimal or entirely absent clinical and field evaluations [42]. Furthermore, these commercial kits are rarely subjected to independent, comparative peer reviews against established in-house protocols, underlining the need for further comparative studies before widespread diagnostic adoption can be recommended. Additionally, high procurement costs and limited reagent shelf-life frequently restrict their implementation in resource-limited settings [5]. To overcome these economic and diagnostic constraints, future efforts should focus on the development of open-source, in-house multiplex real-time PCR assays, which would capitalize on multi-locus targeting to support interpretation of potentially discordant molecular findings [43]. Furthermore, the results of this study demonstrate that when evaluating molecular detection rates using PCR assays, a distinction should be made between the 24 samples with sequence-confirmed pathogenic *Leptospira* DNA and the nine additional conventional PCR-positive findings in MAT and commercial qPCR-negative dogs that lacked reliable sequence confirmation. Such unconfirmed molecular amplifications should be interpreted with caution and categorized separately to avoid overestimating the number of samples with sequence-confirmed pathogenic *Leptospira* DNA.

To our knowledge, this is the first study to provide a genetic characterization of *Leptospira* species in Bosnia and Herzegovina. The *rrs* (16S rRNA) and *secY* genes were strategically selected for phylogenetic analysis due to their established robustness in the molecular typing of leptospires [7]. Sequencing of these loci from 24 selected urine samples confirmed that all the recovered sequences belonged to the pathogenic clade. While the *rrs* sequences clustered within the pathogenic group and shared high identity with the *L. interrogans* complex, the limited sequence diversity of this short fragment precluded definitive species-level identification for all samples. In contrast, the *secY* gene offered superior taxonomic resolution; among the 12 sequences successfully analyzed, 11 demonstrated > 99% identity with *L. interrogans,* while one was identified as *L. borgpetersenii*. These findings reaffirm that while the *rrs* locus is highly suitable for initial differentiation among pathogenic, intermediate, and saprophytic clades, *secY* provides the necessary discriminatory power for precise species identification [7,15].

The predominance of *L. interrogans* among the successfully characterized sequences in our study aligns with global data establishing it as the most prevalent pathogenic species detected in canine populations worldwide [4,28,44,45]. Epidemiologically, this dominance may be linked to the superior environmental persistence of *L. interrogans,* which facilitates indirect transmission, whereas *L. borgpetersenii* relies more heavily on direct host-to-host contact for dissemination [46]. Although *L. borgpetersenii* is less frequently identified in dogs, its role as a pathogen capable of causing severe clinical leptospirosis in canines should not be overlooked [30,32,47].

Given that the exact onset of infection was unknown and that the diagnostic sensitivity and specificity of MAT and PCR heavily depend on the clinical stage of leptospirosis, a parallel serological evaluation of all 124 dogs was conducted. Since the current study relied on convenience sampling, the observed findings reflect the examined cohort rather than representative population prevalence. Using MAT, anti-*Leptospira* antibodies were detected in 20.97% (26/124) of dogs in the primary analytical cohort. This seroprevalence rate aligns closely with the 15.00% serological exposure previously reported in asymptomatic dogs from this region [21]. However, it is significantly lower than the historical seropositivity recorded immediately after the major floods of 2014 (81.25%) and two years thereafter [25]. Variations in serological exposure across these studies may stem from differences in sample size, temporal and geographical sampling boundaries, rainfall volume, and fluctuating ecological or epizootiological conditions. In the present study, while the majority of seropositive dogs reacted to a single serovar, 46.15% exhibited multi-serovar reactivity. This pattern likely reflects cross-reactivity, sequential historical exposure, or contact with antigenically related strains, rather than active multi-serovar co-infection [6]. Consequently, MAT results can only reliably indicate the dominant circulating serogroups; precise serovar and species designation requires definitive serotyping and molecular characterization of isolates, respectively [5]. Consistent with prior regional data, seroreactivity was dominated by the Australis and Pomona serogroups. The detection rates for serovars Icterohaemorrhagiae, Grippotyphosa, Canicola, and Sejroe were comparable to previous findings, with an absolute absence of reactivity against Bataviae, Hardjo, and Autumnalis [21]. This diverse serogroup profile suggests possible exposure of stray dogs to pathogenic leptospires from multiple environmental or wildlife reservoirs. Furthermore, the discrepancies observed between the molecularly identified species and MAT seroreactivity in individual animals reinforce the axiom that infecting species or serovars cannot be definitively inferred solely from MAT results [5]. Comparative Kappa statistics between qPCR and MAT revealed fair agreement (κ = 0.30), underlining that serology alone is insufficient for identifying dogs with detectable *Leptospira* DNA in urine. In our study, only 13 out of the 31 commercial qPCR-positive dogs exhibited positive MAT titers, a discrepancy consistent with previous literature demonstrating the limitations of MAT in identifying dogs with *Leptospira* DNA detectable in urine [17,18,21,28,29,34,42]. By combining serological and molecular methods, the obtained results reflect cumulative evidence of serological exposure and/or molecular detection rather than the prevalence of active infection. The commercial assay yielded a higher positivity rate than MAT when utilized as a single screening strategy, underscoring the advantage of molecular tools in identifying asymptomatic dogs with detectable *Leptospira* DNA in urine [3,35]. Crucially, these commercial qPCR-positive yet MAT-negative dogs may remain undetected by routine serological screening. While the single-timepoint sampling design of this study precludes assessing the duration of bacterial shedding, these asymptomatic dogs may represent a potential source of environmental contamination, although bacterial viability and persistent shedding were not established [18,29,34].

These findings demonstrate serological evidence of exposure and molecular detection of *Leptospira* DNA among the examined stray dogs, supporting the need for further epidemiological investigation in the study area. Epidemiologically, although molecular detection rates in this study represent a snapshot due to the single-timepoint urine collection, the presence of dogs with detectable *Leptospira* DNA in urine suggests a potential risk of environmental contamination. The potential epidemiological significance of urinary DNA detection depends heavily on the bacterial load in the urine, the duration of excretion, and the environmental persistence of the specific *Leptospira* strains [48]. The detection of pathogenic *Leptospira* DNA in urine from stray dogs may reflect exposure through contact with diverse environmental reservoirs and wildlife. Notably, a high *Leptospira* seroprevalence of 34.82% was previously documented in the local fox population [25], emphasizing the interconnectedness of sylvatic and domestic transmission cycles and highlighting the need for expansive, multi-species epizootiological surveys.

Although leptospirosis outbreaks sporadically affect animals and humans in the Republic of Srpska, the true disease burden remains largely obscured due to the historical absence of structured control initiatives and active surveillance programs. To rectify this gap and mitigate the risks to veterinary and public health, the implementation of an integrated animal, environmental, and human surveillance framework under the “One Health” paradigm is highly recommended. Strengthening regional diagnostic capacities through affordable, accessible PCR platforms is an essential first step to overcome current diagnostic limitations that lead to under-reporting and systemic neglect. Ultimately, deploying advanced molecular tools capable of both sensitive detection and high-resolution strain typing is paramount. Such diagnostic upgrades are crucial not only for comprehensive epidemiological mapping but also for evaluating the clinical efficacy of antimicrobial therapies aimed at eradicating renal tubular carriage in PCR-positive animals.

The 11 previously characterized samples were intentionally selected based on commercial qPCR positivity in the earlier study, and their inclusion in the primary epidemiological or inter-assay agreement analyses would introduce selection bias. Accordingly, these samples were analyzed separately as a positive-enrichment panel, while all primary statistical comparisons were restricted to the 124 previously unpublished dogs. Despite the valuable insights gained from this study, several limitations must be acknowledged. Regarding the study population and sampling design, the cohort represents a convenience sample collected over several years rather than a randomized population survey. Consequently, the pooled detection percentages reflect the findings within this specific cohort and should not be generalized as representative prevalence estimates for the wider stray dog population. Furthermore, incomplete standardized records regarding sampling locations and urine collection methods (free-catch versus catheterization) limited the assessment of potential differences associated with these factors. Additionally, the previous leptospirosis vaccination history of these free-roaming dogs remains unknown, which may influence the interpretation of MAT results. Finally, because samples were evaluated at a single timepoint, the PCR findings demonstrate the presence of *Leptospira* DNA but cannot establish bacterial viability or persistent urinary shedding; however, they may indicate a potential epidemiological risk for environmental contamination. These epidemiological constraints are further compounded by limitations in the molecular and microbiological analyses. Specifically, high Ct values compromised our ability to obtain high-quality sequences for phylogenetic analysis. Moreover, the absence of whole-genome sequencing data due to low DNA concentration, combined with the lack of capacity for culture isolation, restricted our capacity to fully elucidate the genetic diversity and evolutionary relationships of the circulating *Leptospira* species. These molecular challenges underscore the need for further molecular and microbiological investigations to improve genomic characterization and surveillance of *Leptospira* in the region.

## 5. Conclusions

This study demonstrates that the comparative use of molecular assays with defined and undisclosed genetic targets provides complementary findings for the investigation of *Leptospira* DNA in urine and helps identify discordant and potentially non-specific amplification results. Furthermore, our findings suggest that combining serological exposure assessment (MAT) and molecular techniques provides a far more comprehensive evaluation of the serological and molecular findings within the examined cohort, capturing both past exposure and the presence of *Leptospira* DNA in urine at the time of sampling better than either method used in isolation. While the combined approach substantially enhances our understanding of the serological and molecular findings in the examined cohort, unconfirmed molecular amplification should be interpreted with caution and categorized separately to avoid overestimating the number of sequence-confirmed infections. The molecularly confirmed detection and phylogenetic analysis of *L. interrogans* and *L. borgpetersenii* DNA in the urine of clinically healthy stray dogs provide the first sequence-based molecular characterization of these pathogenic *Leptospira* species in Bosnia and Herzegovina. This highlights a noteworthy presence of dogs with detectable pathogenic leptospiral DNA at the time of sampling, suggesting a potential risk of environmental contamination, although bacterial viability and persistent urinary shedding were not established. Ultimately, these insights underscore the importance for local public health authorities to implement structured surveillance strategies under the “One Health” framework, focusing on responsible stray dog population management and public education to reduce the potential risk of transmission of this critical zoonosis.

## Figures and Tables

**Table 1:** Primer and probe sequences used for the molecular detection of *Leptospira* spp.

Primers or Probe	Sequence (5′-3′)	Target Gene	Product Size (bp)	Reference
LeptoA LeptoB	GGCGGCGCGTCTTAAACATG TTCCCCCCATTGAGCAAGATT	*rrs* (16S rRNA)	331	[11]
G1 G2	CTGAATCGCTGTATAAAAGT GGAAAACAAATGGTCGGAAG	*secY*	285	[12]
B64-I B64-II	CTGAATTCTCATCTCAACTC GCAGAAATCAGATGGACGAT	*flaB*	563	[12]
LipL32-45F LipL32-286R LipL32-189P	AAG CAT TAC CGC TTG TGG TG GAA CTC CCA TTT CAG CGA TT FAM-AA AGC CAG GAC AAG CGC CG-BHQ1	*lipL32*	242	[13]

**Table 2:** Serogroups, serovars and serovar strains used for MAT.

Species	Serogroups	Serovar (s)	Strains
*L. interrogans*	Pomona	Pomona	Pomona
*L. interrogans*	Icterohaemorrhagiae	Icterohaemorrhagiae	RGA
*L. interrogans*	Canicola	Canicola	Hond Utrecht IV
*L. interrogans*	Australis	Australis	Ballico
		Bratislava	Jez
*L. interrogans*	Autumnalis	Autumnalis	Akiyami A
*L. kirschneri*	Grippotyphosa	Grippotyphosa	Moskva V
*L. interrogans*	Bataviae	Bataviae	Swart
*L. borgpetersenii*	Sejroe	Sejroe	M84
		Hardjo	Hardjobovis

**Table 3:** Comparative serological and molecular results of samples showing at least one positive result in dog sera and urine.

Sample No.	MAT (Serovar/Titer)	^1^ qPCR Commercial Assay (Ct)	*rrs* PCR Assay	mPCR Assay	*lipL32* qPCR Assay (Ct)	Sequencing (Gene/Species Identification)
***A. Primary cohort (n = 124)***						
22	Aus 200	-	-	-	-	NS
23	Gripp 100	-	-	-	-	NS
25	Canic 200; Ictero 100	-	-	-	-	NS
28	Sejr 100	-	-	-	-	NS
33	-	- (44.97) ^s^	+	-	-	rrs **
37	Pom 400	30.15	+	-	31.85	*rrs*; LI
38	Pom 200	-	-	-	-	NS
40	-	-	+	-	-	rrs *
42	Pom 800; Aus 100	-	-	-	-	NS
45	Aus 100	-	-	-	-	NS
49	*-*	-	-	+	-	secY *
52	*-*	33.27	+	+	34.32	*secY; rrs*; LI
55	Pom 100	29.85	+	+	31.28	*secY*; LI
57	-	31.60	+	+	33.82	NS
59	Aus 400; Brat 100	34.69	+	+	37.03	*secY; rrs*; LI
60	-	31.11	+	+	36.28	*secY; rrs;*LI
61	-	-	+	-	-	NS
64	Ictero 200; Canic 200	34.95	+	+	36.18	*rrs*; LI
66	-	32.76	+	-	-	NS
67	Pom 400	-	-	-	-	NS
68	-	38.83	+	-	-	NS
70	-	35.06	+	-	-	*rrs*; LI
72	Canic 200; Aus 100	-	-	-	-	NS
73	-	34.91	+	-	37.45	*rrs*; LI
74	Aus 800	30.24	+	+	33.65	*secY*; LI
80	Aus; Gripp; Canic 100	35.16	+	+	33.52	*secY;* LB
85	Pom 100	30.77	+	+	34.48	NS
87	-	35.32	+	+	34.66	*secY*; LI
88	-	35.35	+	+	35.05	*secY*; LI
89	-	34.52	+	+	33.43	*secY*;LI
90	-	28.11	+	+	32.87	NS
91	-	33.77	+	+	31.54	*rrs*; LI
94	Pom 100	33.04	+	+	30.94	*rrs*; LI
95	Aus 1600; Brat 800; Pom 200; Ictero 200	33.50	+	+	33.81	*secY*; LI
98	Aus 1600; Brat 800	28.00	+	+	25.40	*secY*; LI
99	-	36.74	-	-	-	NS
100	-	37.20	-	+	-	NS
103	Pom 800	38.08	-	+	-	NS
104	-	37.28	+	-	31.67	NS
107	Aus 100; Ictero 100;	-	-	-	-	NS
108	Aus 100	-	-	-	-	NS
111	-	-	+	-	-	NS
112	-	-	+	-	-	NS
115	Aus 400; Ictero 200	38.34	-	-	36.25	NS
116	Ictero 400	-	-	-	-	NS
117	-	-	+	-	-	rrs *
120	-	36.87	+	-	35.94	NS
125	-	36.97	+	-	36.32	NS
127	-	34.32	+	+	33.66	NS
128	Aus 400; Pom 200; Ictero 100; Brat 100	30.20	+	+	32.30	NS
131	Gripp 400; Pom 100	-	-	-	-	NS
132	-	-	+	-	-	NS
135	-	-	-	+	-	*secY* **
***B. Previously characterized positive-enrichment panel (n = 11)***						
**Sample No.**	MAT (Serovar/titer)	^1^ qPCR commercial assay (Ct)	*rrs* PCR assay	mPCR assay	*lipL32* qPCR assay (Ct)	Sequencing (Gene/Species identification)
1	-	36.76	-	+	-	NS
2	-	37.85	-	-	-	NS
3	-	34.95	+	-	35.17	*rrs*; LI
4	-	33.76	+	-	-	*rrs*; LI
5	-	33.91	+	-	-	*rrs*; LI
6	Pom 400; Aus 100	- (44.34) ^s^	-	-	-	NS
7	-	32.90	+	+	33.40	*rrs*; LI
8	-	30.85	+	+	32.10	*rrs*; LI
9	-	33.63	-	-	-	NS
10	*-*	29.38	+	+	31.20	*secY*; LI
11	*-*	33.73	+	+	34.67	*rrs*; LI

Cohort classification: Part A presents the dogs with at least one positive serological or molecular finding from the primary cohort of 124. Part B presents 11 previously characterized samples selected on the basis of commercial qPCR results in the previous study. “+”: positive result; “-”: negative result. Abbreviations: Ictero, Icterohaemorrhagiae; Brat, Bratislava; Sejr, Sejroe; Pom, Pomona; Aus, Australis; Gripp, Grippotyphosa; Canic, Canicola; LI, *Leptospira interrogans* group; LB, *Leptospira borgpetersenii*; NS, not sequenced; * Represents a non-specific sequencing result; ** indicates that a sequence of unsatisfactory quality was obtained (unreliable for species identification). ^1^ qPCR, commercial qPCR assay; ^s^ Ct values > 40.00 were considered borderline according to the manufacturer’s instructions but were classified as negative for statistical and comparative analyses in this study. Sample No. 33, from the primary cohort, yielded a Ct value of 44.97. Separately, sample No. 6, included in the previously characterized panel, had yielded a positive commercial qPCR result in the previous study [21]. Upon retesting in the present study, this sample yielded a Ct value of 44.34.

**Table 4:** Positive, negative, and overall percentage agreement of the molecular assays.

Assay Comparison	PPA ^1^ (%)	NPA ^2^ (%)	OPA ^3^ (%)	Cohen’s (k)	McNemar *p*
Comparison with the qPCR ^4^					
*rrs* PCR vs. qPCR ^4^	87.10	92.47	91.13	0.77	0.5488
mPCR vs. qPCR ^4^	67.74	97.85	90.32	0.72	0.0386
*lipL32* qPCR vs. qPCR ^4^	80.65	100.00	95.16	0.86	0.0313
Pairwise comparison of the other molecular assays					
*rrs* PCR vs. mPCR	-	-	84.68	0.57	0.0192
*rrs* PCR vs. *lipL32* qPCR	-	-	91.13	0.76	0.0117
mPCR vs. *lipL32* qPCR	-	-	91.94	0.74	0.7539

^1^ PPA—Positive Percentage Agreement; ^2^ NPA—Negative Percentage Agreement; ^3^ OPA—Overall Percentage Agreement; qPCR ^4^—commercial qPCR.

## Data Availability

The original contributions presented in this study are included in the article. Further inquiries can be directed to the corresponding authors.

## Abbreviations

The following abbreviations are used in this manuscript:

Abbreviation	Definition
MAT	Microscopic agglutination test
PCR	Polymerase chain reaction
qPCR	Quantitative polymerase chain reaction
mPCR	Multiplex polymerase chain reaction
DNA	Deoxyribonucleic acid
Ct	Cycle threshold
CI	Confidence interval
PPA	Positive Percentage Agreement
NPA	Negative Percentage Agreement
OPA	Overall Percentage Agreement
